# The Ketogenic Diet in the Treatment of Post-concussion Syndrome—A Feasibility Study

**DOI:** 10.3389/fnut.2020.00160

**Published:** 2020-09-10

**Authors:** Michael A. Rippee, Jamie Chen, Matthew K. Taylor

**Affiliations:** ^1^Department of Neurology, University of Kansas Medical Center, Kansas City, KS, United States; ^2^Center for Concussion Management, University of Kansas Health System, Kansas City, KS, United States; ^3^Department of Dietetics and Nutrition, University of Kansas Medical Center, Kansas City, KS, United States; ^4^Alzheimer's Disease Center, University of Kansas, Fairway, KS, United States

**Keywords:** mTBI, post-concussion syndrome, ketogenic diet, ketones, cognition

## Abstract

Concussion is the most common form of mild traumatic brain injury (mTBI). Although most patients' symptoms resolve within a month, patients with post-concussion syndrome (PCS) may continue to experience symptoms for years and have limited treatment options. This pilot study assessed the feasibility and symptom-related effects of a ketogenic diet (KD) in patients with PCS symptoms. The Ketogenic Diet in Post-Concussion Syndrome (KD-PCS) was a single-arm trial of a 2-month KD high in non-starchy vegetables and supplemented with medium-chain triglyceride (MCT) oil. Macronutrient targets were ≥70% fat, ≤10% carbohydrate, and the remainder as protein as energy. We assessed feasibility by daily self-reported measure of urine acetoacetate and collection of 3-day food records and serum beta-hydroxybutyrate at multiple timepoints. We assessed symptoms by administering the Immediate Post-Concussion Assessment and Cognitive Testing (ImPACT) and Modified Balance Error Scoring System (M-BESS) at baseline and month 2 and the Post-Concussion Symptom Scale (PCSS) at baseline, month 1, and month 2. Fourteen participants enrolled in the KD-PCS. Twelve participants completed the study and 11 implemented the KD (73% fat, 9% carbohydrate, and 18% protein) and achieved ketosis. One participant complained of MCT-related diarrhea that resolved and another reported nausea and fatigue that resulted in withdrawal from the study. Among compliant participants, the visual memory domain of the ImPACT improved by 12 points (*p* = 0.02) and PCSS scores improved by 9 points, although not statistically significant. This pilot trial suggests that the KD is a feasible experimental treatment for PCS and justifies further study of its efficacy.

## Introduction

Concussions are the leading form of mild traumatic brain injury (mTBI). The CDC reported the U.S. population sustains 1.7 million concussions annually and, although underreported by as much as 80%, the annual rate of concussion has more than doubled in the past decade (1). Evidence-based clinical management of concussions, therefore, is of particular relevance. While 90% of patients have resolution of symptoms within 4 weeks, the remainder experience persistent and oftentimes debilitating symptoms for months or even years (2), a condition termed post-concussion syndrome (PCS) (3). Unresolved cognitive or memory problems can significantly limit daily activities and have potentially neurodegenerative consequences later in life (4). Currently, treatment is aimed at symptoms but rarely addresses the underlying injury. This is an important limitation in treating post-concussion syndrome and can lead to prolonged recovery periods.

The brain is a highly metabolic organ that requires substantial and uninterrupted energy, usually supplied by glucose. Glucose uptake is severely diminished in the metabolic cascade that occurs after a concussion injury (5), resulting in a condition of brain hypometabolism that can persist for months (6). Correction of this bioenergetic deficit may serve as a potential therapeutic target for underlying post-concussive symptoms. One such approach, the high fat, carbohydrate restricted ketogenic diet (KD), reduces reliance upon glucose metabolism in favor of metabolism of ketones, the body's alternative energy substrate. Ketones contribute significantly to cerebral metabolism (7–9) which, perhaps most importantly, appears to remain intact in conditions with impaired glucose metabolism (10–12).

The KD is used effectively for children with intractable seizures (13–16) and has gained significant interest as a possible therapeutic approach in other neurological conditions with a growing number of studies demonstrating a potential neuroprotective role (17–20). Our recent data also suggests that the KD offers symptomatic benefit in patients with Alzheimer's disease (AD) (21), a condition with a similar observance of hypometabolism due to decreased brain glucose consumption (22, 23). In AD, it is suggested that raising levels of serum beta-hydroxybutyrate (BHB), the primary circulating ketone body, to 0.5 mmol/L is sufficient to bolster brain metabolism (24). KDs and circulating ketones also have purported signaling properties (25), reduce oxidative stress (17, 26, 27), decrease systemic inflammation (28), and promote mitochondrial function (29). In animal TBI models, ketosis-inducing nutritional approaches have been demonstrated to have neuroprotective benefit (30–35) and promising improvement in cognition and behavior (31, 32). Acutely post-TBI, cerebral ketone concentration is elevated and can be modulated through administration of ketosis-inducing nutrition (36). Elevated cerebral ketone concentration could be indicative of a metabolic adaptation to ketone metabolism in the early stages of TBI. Given the existing mechanistic evidence, human clinical investigation into the KD's potential as a therapy in TBI is warranted.

The primary purpose of the present study was to establish feasibility of implementing an 8-week KD in patients with PCS symptoms. Our secondary aims were to assess changes in cognition and self-reported symptoms coincident with the KD treatment. We hypothesized that patients would be compliant with the 8-week KD protocol, identified by monitor of dietary intake and two measures of ketosis, and that compliant participants would experience improvement in cognitive performance and symptomology.

## Methods

The Ketogenic Diet in Post-Concussion Syndrome (KD-PCS) was a single-arm, pilot clinical study with a target enrollment of 14 subjects with Post-Concussion Syndrome (PCS). The protocol required participants to maintain an MCT supplemented KD for 8 weeks. The KD was isocaloric and increased dietary fat to offset decreased carbohydrate consumption, thus we refer to our diet as a very high-fat KD (VHF-KD) to distinguish our diet from a calorie-restricted, low carbohydrate KD.

We prospectively recruited participants from The Center for Concussion Management clinics in the Neurology and Trauma Surgery Departments at The University of Kansas Health System.

### Participants

Participants all had PCS defined as having suffered a concussion resulting from a direct blow, rotation, or whiplash injury to the head or body. The subjects had to be at least 4 weeks post-injury when recruited and have persistent symptoms that included cognitive problems (slowed processing, fogginess, confusion, impaired short-term memory, limited attention span, poor problem solving) as assessed by self-report, clinical observation, or neurocognitive testing. Individuals were required to have a body mass index (BMI) of >21 kg/m^2^ and have mastery of the English language. Only patients between the ages of 18–65 years old were considered for participation. A complete metabolic panel which included electrolytes, liver function tests (LFTs), and blood glucose levels, hematology, insulin, beta-hydroxybutyrate, and cholesterol levels were collected and reviewed after consent but resulting lab values did not determine eligibility. Exclusion criteria included serious medical risks such as insulin dependent diabetes mellitus, ongoing cancer, or recent vascular event (i.e., stroke, heart attack, angioplasty, etc.), neurologic conditions other than mTBI (including cognitive dysfunction), consumption of greater than two alcoholic drinks per day (as determined by patients' self-report), and individuals involved in a lawsuit related to their injury (37). The KU Medical Center's Institutional Review Board approved the protocol. Informed consent was obtained from all study participants as per institutional guidelines.

### Dietary Intervention

Participants received nutrition counseling from the study registered dietitian (RD) at the baseline study visit. They were counseled to consume a self-selected, 1:1 ratio (ratio of grams of lipid to grams of non-lipid) VHF-KD in which energy was derived of 5–10% carbohydrate, 70–75% fat, and 20–25% protein. Dietary principles of the VHF-KD have been previously described (38) and are presented in Table 1. Participants were encouraged to consume a medium chain triglyceride (MCT) oil (NOW Foods, USA) containing a combination of C8:0 and C10:0 fatty acids. To promote MCT oil tolerance, dosage started at 1/2 tablespoon/day for the first week and increased by 1/2 tablespoon weekly until reaching a goal of 1–2 tablespoons/day based on individual tolerance. Energy needs were determined using the Mifflin-St Jeor equation (39). Weight loss was discouraged in this study, thus energy needs were adjusted as needed during the intervention to prevent excessive weight loss among participants. Provided materials included a VHF-KD diet manual containing a description of dietary principles and sample recipes, a 2-month supply of MCT oil, and a daily multivitamin (Kirkland Signature, USA) to address potential micronutrient deficiency concerns.

**Table 1:** KD-PCS ketogenic diet education principles.

**Component**	**Guidelines**
**Macronutrient**	
Fat	Consume at least 70% as energy
	Focus: extra virgin olive oil, avocado, olives, nuts and seeds
	Moderate: nut and seed butters, butter, bacon
Carbohydrate	Restrict to 10% of energy or less
	Primarily comprised of non-starchy vegetables
Protein	Not to exceed 100 g for most individuals
	Focus: fatty fish, eggs, dark meat poultry, unprocessed red meat
	Limit: processed meat
**Food groups**	
MCT oil	Titrate dosage weekly by 1/2 tablespoon starting at 1/2 tablespoon per day
	Aim for 1–2 tablespoons daily, as tolerated
	Best tolerated in coffee and blended with additional long-chain fat source
Vegetables	Unlimited intake of non-starchy vegetables
	Eliminate starchy vegetables
	Add fat prior to and after cooking vegetables
Fruit	Restrict to 1/4–1/2 cup of berries per day
	Unlimited avocado intake
Dairy	Heavy cream as an additive to coffee or tea
	1-2 servings of full fat cheese allowable
	Limit unsweetened, full fat milk or yogurt to no more than 1/2 cup daily
Grains	Eliminate refined and whole grains
	Replace grain flours with almond or coconut flour
Sweetener	Eliminate sugar intake
	Use sweeteners sparingly, primarily Stevia or Truvia
Water	Drink at least 64 fluid ounces of water daily
**General**	
Supplementation	Multivitamin (not included in this analysis)
Urinary ketones	Measure and record urinary ketones daily
	If not producing ketones, reduce carbohydrate, and increase fat

### Dietary Assessment

Dietary intake was measured using 3-day food records (3DFR). The study RD provided written and verbal instructions to participants to complete the 3DFR. Dietary intake was recorded in real time and included 2 weekdays and 1 weekend day at baseline, month 1, and month 2. The RD reviewed completed food records with participants at study visits to ensure completeness and address detail clarifications. Baseline 3DFR reflected dietary intake prior to initiation of the VHF-KD while 3DFR from month 1 and month 2 reflected intake during the VHF-KD intervention. Food record data were entered into the Nutrition Data System for Research (NDSR) 2016 to quantify food and nutrient intake.

### Biomarker, Safety, and Anthropometric Assessments

Participants self-monitored urine ketones daily, in the early evening, using urine acetoacetate test strips (Ketostix, Bayer, Germany). Daily urine ketone status was recorded as either negative, trace (5–14.9 mg/dL), small (15–39.9 mg/dL), moderate (40–79.9 mg/dL), or large (80+ mg/dL) in a provided diary. Days in which participants did not measure the ketone levels were conservatively tallied as a “negative” ketone response.

All serum biomarker and lab tests were collected after a 12-h fast. Full lipid, hematology, and metabolic panels were collected at the baseline and month 2 (end of diet intervention) visits, and these assays were performed by the Quest Diagnostics clinical laboratory. Serum beta-hydroxybutyrate (BHB) and insulin levels were measured at all visit time points (baseline, month 1, and month 2) by Quest Diagnostics clinical laboratory. Homeostatic model assessment 2-insulin resistance (HOMA2-IR) values for each participant were calculated using a HOMA2 Calculator (v. 2.2.3; University of Oxford, United Kingdom).

Height and weight were measured for all subjects. BMI (kg/m^2^) was calculated using weight and height measurements.

### Dietary Obstacles and Palatability

Participants completed short questionnaires upon study exit. These questionnaires were designed to identify obstacles encountered during the VHF-KD as well as the palatability and pleasure of the food items consumed while on the VHF-KD.

### Cognitive Testing

We assessed cognitive performance utilizing the Immediate Post-Concussion Assessment and Cognitive Testing (ImPACT). The ImPACT test was administered at the study baseline visit as well as at the month 2 visit.

### Symptom Evaluation

Participants completed a Post-Concussion Symptom Scale (PCSS) checklist at all three study visits. The PCSS is a self-reported assessment of 22 symptoms using a Likert-type scale ranging from 0 to 6, with 0 indicating no difficulty with the outlined symptom and ratings of 1–6 representing mild-to-severe difficulty with the symptom. The PCSS is part of the ImPACT computerized testing and was conducted with the ImPACT test at baseline and month 2 visits. A paper version of the PCSS was completed by the subject at all three study visits. We also assessed depression and anxiety symptoms at baseline and at the study conclusion. To assess severity of depression symptoms, participants completed the Patient Health Questionnaire (PHQ-9). The PHQ-9 is a 9-question survey that asks questions about depression symptoms over the previous 2 weeks (40). Each question is scored 0–3 and summed to form a maximum score of 27 with higher scores relating to more severe depression symptoms. We assessed anxiety symptoms using the General Anxiety Disorder (GAD-7) questionnaire, a 7-question, self-reported anxiety survey that has been validated for the general population (41). Each question is scored 0–3 and summed to form a maximum score of 21 with higher scores relating to more severe anxiety.

### Balance Testing

The Modified Balance Error Scoring System (M-BESS) (42) was completed at the baseline and month 2 visits. The M-BESS tests an individual's postural stability on a firm, flat surface in three different stances: double-leg, single-leg, and tandem gait. The test is performed barefoot with eyes closed and hands placed on the hips. Each stance has a maximum score of 10 and 1 point is subtracted for each mistake. Total M-BESS scores are calculated by summing the scores of each stance.

### Statistical Analysis

The primary aim was to report feasibility of the VHF-KD in patients with PCS with a secondary aim of reporting preliminary efficacy. We report continuous data as mean ± SD throughout, but as data were mostly non-normally distributed, continuous variables are presented in tables as median [Q1;Q3]. We described VHF-KD feasibility using descriptive statistics regarding compliance and study withdrawal. All data were determined to be non-normal through the Shapiro-Wilk test for normality and visualization of frequency histograms and normal Q-Q plots. Owing to no missing longitudinal data among study completers, we used the non-parametric Kruskal-Wallis test for variance for all ANOVA models and applied the Dunn's Test of multiple comparisons with no adjustment for data with values at baseline, month 1, and month 2. Statistical analyses were performed using R (v. 3.6.1; R Foundation, Vienna, Austria). Statistical tests were two-tailed, and significance was set at *P* < 0.05.

## Results

Fourteen participants enrolled in the KD-PCS over a course of 18 months. Twelve participants were female and two were male with a mean age of 45.3 ± 12.2 years (median: 51.0, IQR: 39.0–57.5). To meet our enrollment target, we screened 283 charts of patients that presented to the KU Health System Concussion Clinic. The study coordinator approached 39 of these patients at an in-person clinic visit to consider participation in this study. Supplemental Figure 1 depicts recruitment and participant flow.

Twelve participants completed the study. Two participants withdrew from the study after completion of the baseline study visit—one withdrew due to complications with dietary adherence and the other participant was lost to follow-up prior to the month 1 visit. One participant that completed the study was not compliant with the diet, failing to reach ketosis neither by serum measure nor urinary measure. The remaining 11 participants were successful at implementing the VHF-KD intervention (79% compliance rate) by registering elevated serum BHB and urinary ketone measures and were included in the analyses of outcomes.

**Figure 1:**
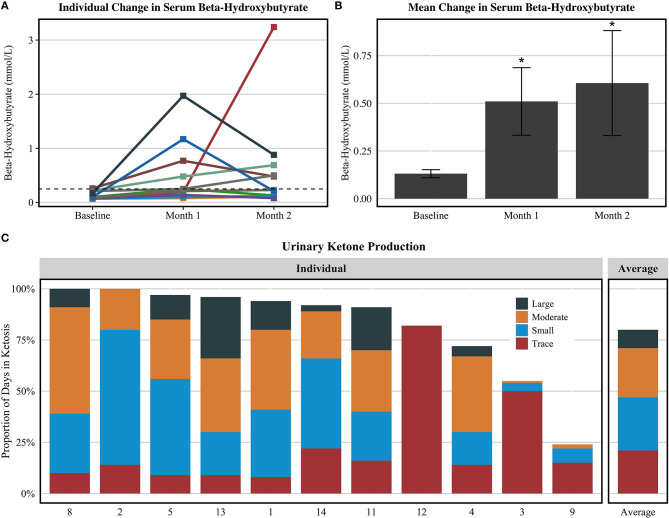
Urine acetoacetate and serum BHB values among compliant participants (*n* = 11). **(A)** Serum BHB values for each subject. **(B)** Mean serum BHB values ± standard error. **(C)** The proportion of days in which each participant's self-reported urine acetoacetate analysis revealed ketosis and depth of ketosis achieved. The figures exclude the one participant that completed the study procedures but did not record elevated ketone bodies at any point during the intervention. *P < 0.05 compared to baseline value via Dunn's test of multiple comparisons.

Participants were not in ketosis at baseline (0.1 ± 0.1 mmol/L) and significantly raised serum BHB levels at month 1 (0.5 ± 0.6 mmol/L) and month 2 (0.6 ± 0.9 mmol/L), illustrated in Figures 1A,B. Considering the 11 compliant participants, self-reported positive urinary ketone production was recorded a mean of 48.0 ± 13.6 days (80% of duration) during the intervention with varying reported depth of ketosis. Of the study duration, ketosis depth as a proportion of days in ketosis was reported as 21% trace, 26% small, 24% moderate, and 9% large. Blood and urinary ketone data are presented in Figure 1.

Dietary intake data from the regular diet and VHF-KD are reported in Table 2. Energy intake was similar between the two diets. During the intervention, total fat intake increased from 96.9 to 145.0 g (*p* < 0.001), total carbohydrate intake was significantly reduced 196.5 to 42.5 g (*p* < 0.001), and protein intake was similar. Participants consumed 73% of fat, 9% of carbohydrate, and 18% of protein as a proportion of energy while on the VHF-KD. Average consumption of MCT oil was 1.5 tbsp/day (21.5 g/day). All micronutrient intake was similar between the two diets except for decreased thiamin and calcium intake on the VHF-KD. Non-starchy vegetables increased from 2.1 servings to 3.1 servings on the VHF-KD (*p* < 0.05).

**Table 2:** Dietary intake on the regular and ketogenic diets (*n* = 11)^a^.

	**Regular diet**	**Ketogenic diet**	***p*-value**
**Energy/Macronutrients**			
Energy, kcal	1918.7 [1767.8;2213.8]	1810.5 [1675.2;1947.8]	0.25
Fat, g	95.0 [86.0;111.9]	145.4 [135.6;158.5]	0.004
% of energy	42.0 [40.5;45.0]	70.5 [66.8;76.3]	0.001
Carbohydrate, g	187.2 [166.6;230.4]	42.1 [32.8;51.6]	0.001
% of energy	37.4 [33.4;43.0]	8.3 [7.0;9.5]	0.001
Protein	75.3 [65.0;81.2]	80.3 [65.5;100.6]	0.64
% of energy	13.4 [12.6;15.9]	19.9 [16.3;21.5]	0.13
**Fatty acid composition**			
MCT Oil, g	0.0 ± 0.0	21.5 ± 6.5	<0.001
SFA, g	32.8 [29.2;39.6]	68.1 [65.0;80.0]	0.003
MUFA, g	33.8 [33.0;44.3]	44.5 [42.2;48.1]	0.13
PUFA, g	20.5 [17.3;21.1]	14.7 [12.0;19.9]	0.36
Trans fatty acids, g	2.1 [1.8;2.8]	3.1 [1.7;3.4]	0.56
Eicosapentanoic acid, g	<0.1 [<0.1; <0.1]	<0.1 [<0.1;0.2]	0.20
Docosahexanoic acid, g	<0.1 [<0.1;0.1]	0.1 [<0.1;0.4]	0.09
Cholesterol, mg	363.6 [284.0;497.8]	666.2 [497.1;754.4]	0.02
**Carbohydrate**			
Sugar, g	78.2 [68.3;100.0]	13.8 [12.7;21.8]	0.002
Total fiber, g	16.8 [13.4;20.1]	11.1 [8.6;15.0]	0.04
Soluble fiber, g	5.5 [5.0;6.5]	3.2 [1.7;5.1]	0.03
Insoluble fiber, g	9.7 [8.2;14.3]	8.1 [6.6;9.9]	0.17
**Decreased micronutrients**			
Thiamin, mg	1.5 [1.2;1.6]	0.6 [0.5;1.0]	0.004
Calcium, mg	773.9 [749.9;969.5]	515.7 [440.0;595.0]	0.003
**Fruits and vegetables^b^**			
Fruit	0.6 [0.1;1.2]	0.2 [0.0;0.3]	0.16
Avocados	0.0 [0.0;0.0]	0.1 [0.0;0.6]	0.13
Non-starchy vegetables	2.0 [1.6;2.4]	2.7 [2.5;3.3]	0.02

a *Values are median [Q1;Q3]. Group differences were assessed by the Kruskal–Wallis test for variance. Significance was set at P < 0.05. All dietary data were derived by 3-d food record at each time point. For both diets, nutrient data were derived by food only. MCT, medium-chain triglyceride; MUFA, monounsaturated fatty acid; PUFA, polyunsaturated fatty acid; SFA, saturated fatty acid*.

b *Fruit and vegetable intake reported as 1/2-cup raw servings and 1-cup cooked servings*.

No adverse events were reported, but two minor diet-related complaints were documented in this study. The first was acute diarrhea in one participant after ingestion of MCT oil. Diarrhea symptoms were alleviated by mechanically blending MCT oil with a long-chain fatty acid (emulsification) prior to consumption, a process purported to improve MCT tolerability (43). One participant reported nausea and fatigue in the weeks following VHF-KD initiation that resolved by discontinuation of the diet. This participant discontinued the diet prior to month 1 and withdrew from the study at that study visit. It is notable that all participants were instructed to mechanically blend MCT oil in beverages with an additional long-chain fat source, primarily butter or cream, prior to ingestion. Using this method, there was no report of common symptoms (i.e., nausea, diarrhea, etc.) related to MCT oil tolerability. Comprehensive labs (Table 3) were monitored throughout the study and were unchanged.

**Table 3:** Participant characteristics and blood-derived data among compliant participants (*n* = 11).

	**Baseline**	**Month 1**	**Month 2**	***p*-value**
**Participant characteristics**				
Age	51.0 [39.0;57.5]			–
Days since concussion injury	103.0 [56.0;217.5]			–
Weight, kg	83.6 [70.8;95.5]	82.6 [68.3;90.2]	81.7 [68.5;89.0]	0.74
BMI	27.0 [25.6;33.5]	26.5 [24.7;31.6]	26.2 [24.5;31.2]	0.64
**Blood biomarkers**				
Albumin, g/dL	4.4 [4.3;4.5]	4.7 [4.4;4.8]	4.5 [4.3;4.7]	0.51
Albumin/Globulin ratio	1.5 [1.4;1.8]	1.8 [1.6;1.9]	1.7 [1.6;2.0]	0.20
Alkaline phosphatase, U/L	68.0 [55.0;75.0]	55.0 [46.2;56.8]	58.0 [47.0;66.0]	0.35
ALT, U/L	19.0 [15.5;25.0]	21.0 [18.2;21.8]	19.0 [16.5;21.0]	0.62
AST, U/L	20.0 [17.5;22.5]	20.0 [18.2;21.8]	19.0 [17.0;20.0]	0.58
Calcium, mg/dL	9.8 [9.7;9.9]	9.9 [9.7;10.0]	9.8 [9.6;9.9]	0.47
Carbon dioxide, mmol/L	28.0 [25.5;28.5]	28.0 [26.0;28.8]	27.0 [25.0;29.0]	0.71
Chloride, mmol/L	102.0 [101.5;105.0]	102.0 [100.2;104.0]	104.0 [99.5;105.5]	0.73
Creatinine, mg/dL	0.8 [0.7;0.9]	0.8 [0.8;0.9]	0.8 [0.8;0.8]	0.67
eGFR, mL/min/1.73m^2^	86.0 [77.0;97.0]	84.5 [81.5;98.5]	87.0 [82.5;104.0]	0.67
Globulin	2.8 [2.5;3.0]	2.5 [2.4;2.9]	2.7 [2.3;2.8]	0.38
Potassium, mmol/L	4.5 [4.4;4.7]	4.3 [4.1;4.4]	4.3 [3.9;4.5]	0.31
Sodium, mmol/L	140.0 [138.5;140.0]	139.0 [139.0;140.5]	139.0 [138.5;140.5]	1.00
Total bilirubin, mg/dL	0.6 [0.5;0.8]	0.6 [0.5;0.6]	0.6 [0.4;0.8]	0.83
Total protein, g/dL	7.3 [7.0;7.5]	7.3 [6.9;7.5]	7.0 [6.8;7.4]	0.71
Urea nitrogen, mg/dL	15.0 [13.0;19.0]	14.0 [10.5;15.8]	15.0 [13.0;16.0]	0.63
White blood cell count, thousand/uL	7.0 [4.7;7.9]		6.6 [4.3;7.5]	0.69
Red blood cell count, million/uL	4.4 [4.3;4.8]		4.3 [4.2;4.5]	0.60
Hemoglobin, g/dL	13.3 [13.2;14.7]		13.4 [12.9;13.8]	0.64
Hematocrit	40.2 [39.2;44.2]		39.7 [38.6;42.1]	0.58
MCV, fL	92.7 [90.3;93.9]		91.2 [88.7;94.4]	0.92
MCH, pg	31.0 [29.6;31.5]		30.6 [30.1;31.4]	0.90
MCHC, g/dL	33.3 [33.2;33.9]		33.2 [32.6;33.7]	0.36
RDW	12.5 [11.9;13.6]		12.9 [12.2;13.1]	0.77
Platelet count, thousand/uL	287.0 [241.5;300.5]		291.0 [237.0;331.5]	0.97
MPV, fL	9.8 [9.8;10.4]		10.9 [10.0;11.1]	0.26
Absolute neutrophils, cells/uL	4350.0 [2816.5;5128.5]		4184.0 [2491.0;5209.0]	0.77
Absolute lymphocytes, cells/uL	1847.0 [1574.5;1894.0]		1754.0 [1526.0;1960.5]	0.97
Absolute monocytes, cells/uL	469.0 [346.0;530.0]		399.0 [344.0;462.0]	0.58
Absolute eosinophils, cells/uL	129.0 [106.5;146.5]		108.0 [91.5;205.0]	0.95
Absolute basophils, cells/uL	49.0 [42.5;56.5]		46.0 [42.5;52.0]	0.65
Neutrophils	65.1 [58.9;67.3]		60.0 [54.3;64.8]	0.49
Lymphocytes	25.9 [24.3;30.4]		29.1 [25.3;34.8]	0.43
Monocytes	7.0 [5.3;7.8]		6.0 [5.7;8.1]	0.84
Eosinophils	2.0 [1.4;2.5]		1.7 [1.4;3.3]	0.77
Basophils	0.7 [0.6;1.0]		0.6 [0.6;1.1]	0.89
**Lipids**				
Total cholesterol, mg/dL	195.0 [164.5;206.5]		188.0 [175.0;208.0]	0.87
Triglycerides, mg/dL	95.0 [71.5;119.5]		102.0 [64.0;109.0]	0.87
HDL cholesterol, mg/dL	74.0 [54.5;83.5]		77.0 [53.5;95.0]	0.82
LDL cholesterol, mg/dL	96.0 [77.0;116.5]		93.0 [81.0;119.0]	0.79
Cholesterol/HDL ratio	2.6 [2.1;3.2]		2.8 [2.0;3.7]	0.92
Non-HDL cholesterol	115.0 [91.5;138.0]		114.0 [94.0;140.0]	0.92
**Blood substrate biomarkers**				
Glucose, mg/dL	85.0 [84.0;94.5]	86.0 [82.5;90.0]	85.0 [80.0;93.0]	0.82
Serum beta hydroxybutyrate, mmol/L	0.1 [0.1;0.2]	0.3 [0.2;0.7]	0.3 [0.1;0.6]	0.01
Insulin, uIU/mL	5.9 [3.2;11.4]	6.0 [4.1;7.4]	4.2 [3.2;8.3]	0.71
HOMA2-IR	0.8 [0.4;1.5]	0.8 [0.5;1.0]	0.7 [0.5;1.1]	0.92

*Values are median [Q1;Q3]. Group differences were assessed by the Kruskal–Wallis test for variance. Significance was set at P < 0.05*.

**Figure 2:**
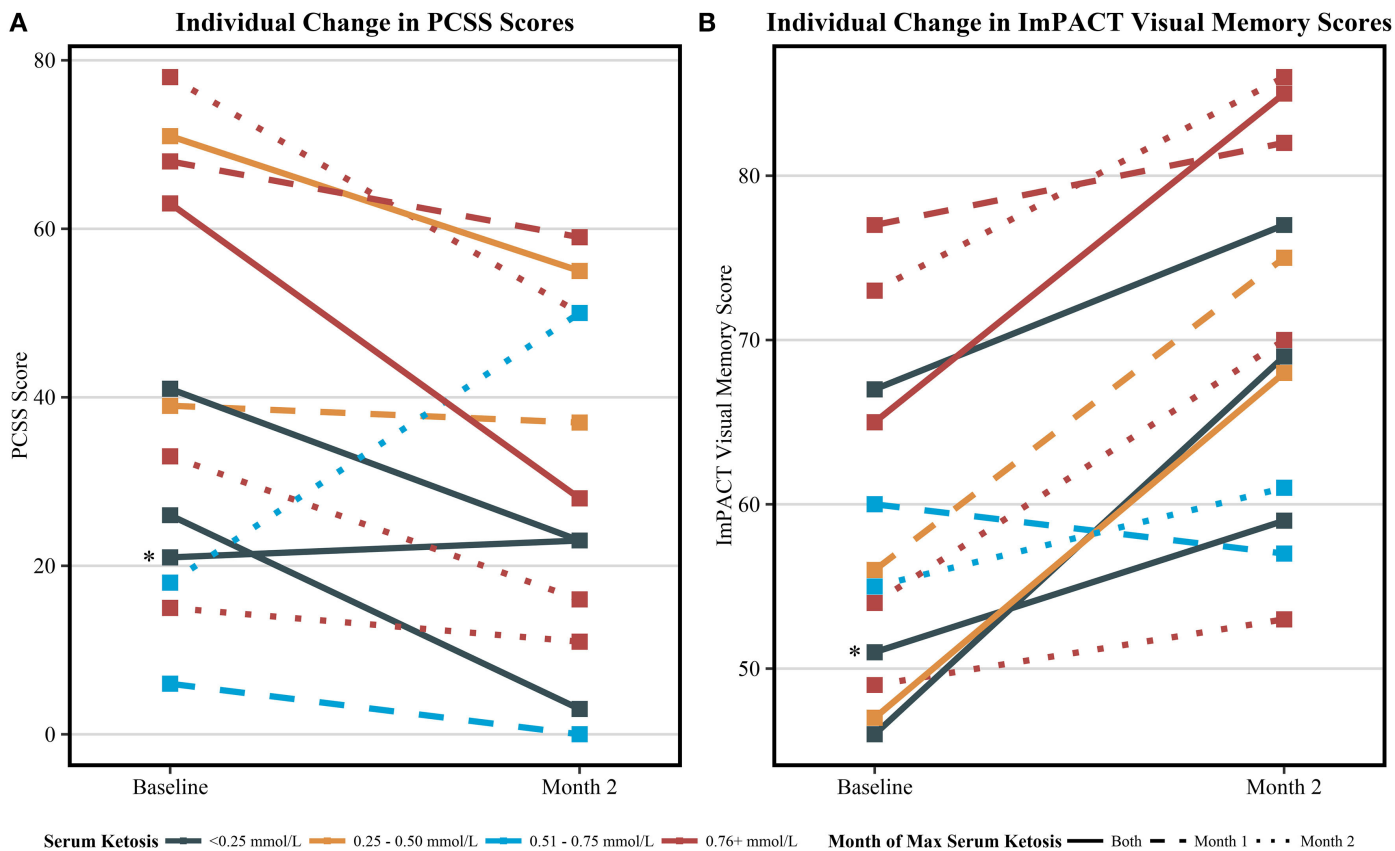
Individual change in symptom and visual memory scores among all completing participants (*n* = 12). **(A)** The individual change in the post-concussion symptom scale (PCSS) from baseline to 2 months. Decreasing scores indicate improvement in self-reported symptoms. **(B)** The individual change in ImPACT visual memory scores from baseline to 2 months. Increasing scores indicate improvement in tested visual memory. Serum ketosis levels were determined by peak measured serum BHB. Different line types indicate the month which peak ketosis was reached, a solid line indicating both month 1 and 2, a dashed line indicating month 1, and a dotted line indicating month 2. One participant (*) was considered not compliant with the KD and did not produce urine ketones as indicated by the urine ketone record nor had elevated serum BHB at any study visits. This participant's symptom score slightly worsened and verbal memory score slightly improved from at the end of the study. The other two participants that did not register elevated BHB at study visits recorded trace ketone production via urine ketone records during the course of the study.

The visual memory domain of the ImPACT assessment improved by a mean of 12.2 points from baseline to month 2 of the intervention (59.0 ± 10.4 vs. 71.2 ± 11.1, *p* = 0.02). Though not significant, mean PCSS scores improved from baseline by 4.6 points at month 1 and 9.6 points at month 2. All assessment results are reported in Table 4. Individual results from the ImPACT visual memory domain and PCSS are presented in Figure 2, stratified by the peak measured serum BHB level achieved during the 2-month study intervention. Baseline scores for visual memory and symptoms were highly variable. Both scores trended toward improved scores for a majority of the individuals except for two; one individual with no detected ketosis via serum or urine had a slightly worsened PCSS score at the end of the intervention and another individual who exhibited elevated serum BHB had significantly worsened PCSS scores and slightly worsened visual memory.

**Table 4:** Cognition, balance, and symptom assessment among compliant participants (*n* = 11).

	**Baseline**	**Month 1**	**Month 2**	***p*-value**
**ImPACT**				
Verbal memory	92.0 [81.5;93.5]		85.0 [81.0;88.0]	0.47
Visual memory	56.0 [51.5;66.0]		70.0 [64.5;79.5]	0.02
Visual motor speed	30.6 [23.7;33.0]		32.9 [26.7;38.7]	0.34
Reaction time	0.9 [0.7;1.0]		0.7 [0.7;0.8]	0.22
**M-BESS**				
Single	3.0 [0.0;4.5]		3.0 [2.0;5.0]	0.43
Double	10.0 [9.0;10.0]		10.0 [10.0;10.0]	0.48
Tandem	5.0 [2.0;7.0]		7.0 [5.0;8.5]	0.18
Total score	17.0 [11.5;20.5]		20.0 [17.5;23.0]	0.29
**PCSS score**	33.0 [19.5;65.5]	34.0 [17.5;56.0]	28.0 [13.5;50.0]	0.34
**PHQ-9 score**	16.0 [8.0;16.5]		8.0 [4.0;11.0]	0.27
**GAD-7 score**	10.0 [3.5;17.5]		7.0 [5.0;10.0]	0.65

*Values are median [Q1;Q3]. Group differences were assessed by the Kruskal–Wallis test for variance. Significance was set at P < 0.05. GAD-7, General Anxiety Disorder 7; ImPACT, Immediate Post-Concussion Assessment and Cognitive Testing; M-BESS, Modified Balance Error Scoring System; PCSS, Post-Concussion Symptom Scale; PHQ-9, Patient Health Questionnaire 9*.

The dietary obstacles and palatability questionnaire was completed by study finishers at the final visit and results are reported in Table 5. Overall, participants reported that they felt adequately trained and confident in following the VHF-KD. No participants reported tolerance issues while following the VHF-KD. “Strongly Disagree” was documented only once on three different statements: “I was able to calculate diet and meals,” “The initial visit with the dietitian was sufficient for initiation,” and “The diet was not too costly.” Four of the 12 study completers reported some level of disagreement that the VHF-KD “was not too costly.” Five participants reported that the VHF-KD improved their symptoms, while the remaining 7 reported that they were unsure whether the VHF-KD had any effect on symptoms.

**Table 5:** Dietary obstacles and palatability among study completers (*n* = 12).

	**Strongly agree**	**Agree**	**Neutral/Unsure**	**Disagree**	**Strongly disagree**
I was able to calculate diet and meals	2 (17%)	6 (50%)	3 (25%)	0 (0%)	1 (8%)
I was able to follow the diet	6 (50%)	5 (42%)	0 (0%)	1 (8%)	0 (0%)
I tolerated the diet	8 (67%)	2 (17%)	2 (17%)	0 (0%)	0 (0%)
The diet improved symptoms	2 (17%)	3 (25%)	7 (58%)	0 (0%)	0 (0%)
I felt adequately trained on the diet	6 (50%)	3 (25%)	1 (8%)	2 (17%)	0 (0%)
The initial visit with the dietitian was sufficient for initiation	9 (75%)	1 (8%)	1 (8%)	0 (0%)	1 (8%)
Follow up with dietitian was adequate	8 (67%)	4 (33%)	0 (0%)	0 (0%)	0 (0%)
I was able to maintain normal activity	10 (83%)	2 (17%)	0 (0%)	0 (0%)	0 (0%)
The diet was not too costly	5 (42%)	3 (25%)	0 (0%)	3 (25%)	1 (8%)
Overall, I was satisfied with the diet	8 (67%)	3 (25%)	1 (8%)	0 (0%)	0 (0%)

*Values are n (%)*.

## Discussion

This is the first study to investigate whether a VHF-KD therapy is feasible in patients with PCS, entailing a 2-month VHF-KD with modest MCT oil supplementation. Our findings suggest that the VHF-KD is feasible in patients suffering from prolonged concussion-related symptoms and has coincident relationship with improvement in visual memory and PCS-related symptomology.

Potential participants were pre-screened and approached during their Center for Concussion Management clinic visits. Out of the 39 patients who expressed willingness to discuss study participation with the study coordinator, 20 requested to consent to enroll in the study. Six of those consenting patients were unable to complete study enrollment and proceed with the baseline visit, either due to personal decision or ineligibility factors. Common recruitment barriers reported by patients to the study coordinator included, but were not limited to, inability or lack of desire to commit to study-related time and diet requirements, fear of blood draws, and active litigation relating to the concussion injury.

The diet proved feasible in 11 of 14 participants (79% compliance) evidenced by intake of the prescribed VHF-KD macronutrient targets and both consistent presence of urinary ketones and elevated blood ketones. Self-reported presence of urinary ketones exceeded our expectations. Eight of the 11 compliant participants reported a frequency of >80% of days in ketosis. During the VHF-KD education portion of the baseline visit, participants were encouraged to achieve a goal of small to moderate ketosis. Ten participants met that goal and 7 exceeded the goal by reporting achievement of large ketosis. It is notable that MCT oil (1.5 tbsp/day) was primarily consumed in the morning with coffee and urine ketone testing occurred in the evening, effectively reducing the potential for artificially large ketosis measurement due to acute MCT-induced elevation in circulating ketones and adequately reflected the ketogenic capacity of the overall diet. Seven of the eleven participants presented with fasted serum BHB levels ≥0.5 mM at a minimum of one of the two study visits concurrent with the VHF-KD intervention. At the month 1 visit, 3 participants with BHB levels <0.3 mM reported having deviated from the VHF-KD in the previous few days due to personal circumstances, such as celebratory events or stress. These events may have affected the potential serum BHB results from this study and suggests a need to incorporate additional blood biomarker measurement in future studies.

Illustrated in Figure 1, participant 9 appeared to have only recorded urine acetoacetate for ~25% of the intervention and did not provide a response for urine ketone measurements 22% of the intervention, which we conservatively interpreted as “negative” readings. However, for the purpose of this feasibility study, we did consider this participant to be compliant with the diet due to elevated serum BHB levels at months 1 and 2 and reported adherence to the target macronutrient composition via 3DFR. Future KD trials should embed strategies within the study design to encourage compliance with collection of ketosis measures to ensure better characterization of KD compliance and determine necessary depth and duration of ketosis for efficacy.

Participants adopted a VHF-KD with a ratio of ~1.2:1 with energy comprised of 73% fat and 9% carbohydrate, slightly exceeding the 1:1 target of 70% fat and 10% carbohydrate. The composition of the VHF-KD consumed in this study is similar in composition to the VHF-KD we previously studied in AD (38). Mean non-starchy vegetable intake increased one full serving while maintaining carbohydrate restriction. We have previously suggested that attention to diet quality may extend the putative benefits of the KD in neurological conditions (44). Non-starchy vegetables are an excellent source of micronutrients, are low in energy density and carbohydrate, and should be consumed in large quantity in a high quality KD.

We also observed improvement in the visual memory domain of the ImPACT test and a 9-point mean improvement in self-report of concussion-related symptoms, an improvement with suggested clinical relevance (45). These improvements should be interpreted with caution given that this was a single-arm study and outcomes measures were secondary aims. Vestibular-ocular impairment is observed post-concussion (46) and may contribute to deficit in visual memory, one of the most observed symptoms in concussion patients which may persist in patients with PCS (47, 48). Due to its consistency as a symptom post-concussion, it is reasonable to hypothesize that the visual memory domain may be one of the most profound to be affected by effective therapies. Our study also complements two animal studies that demonstrated improved cognitive function related to ketone-inducing dietary approaches. Appelberg et al. reported that 7 days post-TBI, rats fed a KD had better improvement than those on a regular diet in motor and cognitive function via the Morris Water Maze task and beam walking assessment (32). This finding was age-dependent as the improvements were observed in rats that received head trauma 35 days post-natal and not in rats that received head trauma 75 days post-natal; an effect speculatively due to resilience to energy metabolism deficit and quicker conversion to ketone metabolism in young vs. old rats (33). The second study demonstrated that rats fasted for 24 h immediately post-concussion had elevated ketone bodies at 24 h, performed better on the Morris Water Maze task at 10 days, and had more cortical tissue at 15 days (31). Other studies suggest that acutely post-TBI, adolescent rats fed a KD have improved cortical contusion volume 1 week post-injury (49) and reduced anxiety and depression symptoms (50).

PCS is still not well-understood. The condition itself is defined by concussion-related symptoms that persist beyond the course of normal concussion recovery, commonly persisting beyond improvement of physiological biomarkers of existence of mTBI (3). Acutely after mTBI, cerebral perfusion (51–53) and glucose metabolism become impaired (54). This hypometabolic state is typically corrected within a few days to a week; however, limited evidence suggests chronic impairment of these mechanisms as a compelling explanation for sustained symptoms. Under normal conditions, cerebral blood flow (CBF) is coupled with the cerebral metabolic rate of glucose to meet brain energy demand. Immediately following mTBI, CBF decreases (55) followed by hyperemia and later uncoupling of CBF and cerebral metabolism (56) that persists in those with PCS (57). The metabolic implications of this cascade leading to chronic reduction in brain glucose metabolism may be an underlying cause of symptoms in patients with PCS (58). If so, PCS shares a similar hallmark with AD (59), a condition in which ketone metabolism mechanisms and potential therapeutic value of the KD have recently been studied with some extensity (10, 21, 24, 60). It is possible that AD and PCS share the common thread of underlying cerebral glucose hypometabolism, potentially rescuable through exchanging glucose metabolism in favor of ketone bodies and its downstream metabolic effects (28). We are unable to assess mechanisms through which the KD may influence cognition or symptom change in this study.

We also consider other mechanisms by which the KD and ketone bodies, independently, may be beneficial in PCS (44). Ketone body metabolism is suggested to produce fewer reactive oxygen species (ROS) than glucose metabolism and KDs, specifically, have been shown to upregulate mitochondrial antioxidant systems that scavenge ROS. Reduction in ROS activity may reduce ROS-related mitochondrial damage and preserve mitochondrial function. In a ketotic state, mitochondrial uncoupling proteins (UCP) are upregulated, enhancing energy production derived by the electron transport chain (ETC). Evidence also suggests that the KD upregulates the anti-inflammatory pathway, nuclear factor-E2 (Nrf2). Through both direct effects and preserved mitochondrial function, KDs and ketone bodies may also improve regulation of brain neurotransmitter levels. Considering the evidence for KDs and ketone bodies, it would be desirable to know whether potential benefit in patients with PCS would require adherence to the KD or if other ketone-generating approaches, such as MCT or exogenous BHB supplements alone, are sufficient.

Our group's previous success with implementing the VHF-KD in an AD clinical trial (21) provided preliminary experience with and adaptability to the potential challenges faced at both the individual and systemic level in this study. The diet education provided to participants in this study was refined from the previous AD study to portray a simple message and empower participants to follow a VHF-KD that fit their food preferences and proficiency for food preparation. In addition to a 3-day sample menu adjusted to each participant's energy needs, we provided nearly 100 KD-friendly recipes with variable time allocation and skill requirement along with recommended recipe resources. Through this process, we've found that one of the simplest guides for success with the KD is to use Ketostix readings as an immediate feedback mechanism; if no ketone presence is detected, then the participant's actionable step, using the principles of a well-formulated KD, is to simply consume additional fat and further restrict carbohydrate the following day. Contrasting the participants from this study with the previous AD study, compliance is likely more attainable due to patient independence without the need of a study partner and the associated burden for a study partner to implement the diet. On the other hand, some participants in this study required a significant level of contact with the study team to remain compliant with the KD, seemingly due to memory-related symptoms suffered from the patient's concussion.

The KD is most well-known as an effective treatment in refractory epilepsy (13–16). It is important, however, to distinguish the KD used for treatment of epilepsy and other conditions in which the KD may also be valuable as a treatment. Patients with epilepsy use the KD to maintain seizure control and it has been suggested that, in some cases, serum BHB levels must reach ≥4.0 mmol/L for this benefit (61). The KDs in these particular circumstances are strict and require carbohydrate restriction of ≤2% of energy and consumption of ≥90% of fat as energy (44). Compliance with a diet of this macronutrient distribution within free-living adults with a condition of brain bioenergetic disruption is likely not feasible, nor necessary. Patients with AD improve overall cerebral metabolic rate with modest ketosis approaching 0.5 mmol/L (24). If these mechanisms overlap, we hypothesize that the majority of our participants raised their serum BHB to a level that may have been sufficient to contribute as a substrate for brain metabolism.

We also acknowledge limitations that reduce our ability to interpret feasibility and outcomes in this study. Participants performed daily self-monitoring of ketosis by urine acetoacetate strips, which presents two limitations. First, self-report is subject to inaccurate reporting by the participant. Second, although urine acetoacetate measurement is positively associated with blood-derived measurement of ketosis (62), capillary measurement of BHB by fingerstick is a more sensitive measure of circulating ketones and should be considered as a measurement in future KD trials. We also assessed serum BHB levels in the morning after a 12 h fast at only two time points during the intervention. From this limited assessment, we are unable to elucidate whether serum BHB levels may have been highest at this point due to a fasting effect, or whether serum BHB levels may have been higher after consuming the KD due to consumption of fat substrate for ketogenesis. The direction of post-prandial BHB response is influenced by compliance to the KD macronutrient profile. Measurement of capillary BHB at multiple, varying timepoints would provide better insight for characterization of dietary compliance and general ketone status. Finally, this is a small pilot study that aimed to test feasibility of the KD in individuals with PCS. Lacking a control group, we encourage caution in interpreting the results of this study as we are unable to determine if coincident changes in cognition and reported symptoms were due to the KD intervention.

The KD-PCS was designed to assess feasibility of a VHF-KD in patients with PCS and confirms that adherence to a VHF-KD in this population is feasible. Due to the small sample size and single-arm design of the study, we are unable to conclude that the VHF-KD was responsible for observed improvement in visual memory or report of symptoms. We urge caution in interpreting our coincidental findings. However, the results from this study do suggest potential for the KD to benefit patients with prolonged symptoms due to concussion and warrants future studies to investigate the KD as a potential therapeutic approach.

## Data Availability Statement

The datasets generated for this study are available on request to the corresponding author.

## Ethics Statement

The studies involving human participants were reviewed and approved by University of Kansas Medical Center IRB. The patients/participants provided their written informed consent to participate in this study.

## Author Contributions

MR and MT contributed conception and design of the study. JC organized the database. MT performed the statistical analysis and wrote the first draft of the manuscript. MR, JC, and MT wrote sections of the manuscript. All authors contributed to manuscript revision, read the manuscript, and approved of the submitted version.

## Conflict of Interest

The authors declare that the research was conducted in the absence of any commercial or financial relationships that could be construed as a potential conflict of interest.
