# Supplementary figures and images for: The Ketogenic Diet in the Treatment of Post-concussion Syndrome—A Feasibility Study
Source: Front Nutr. 2020 Sep 10;7:160. doi: 10.3389/fnut.2020.00160 (PMC7511571; doi:10.3389/fnut.2020.00160)

**Supplemental Figure 1. CONSORT diagram for KD-PCS recruitment and analysis.**

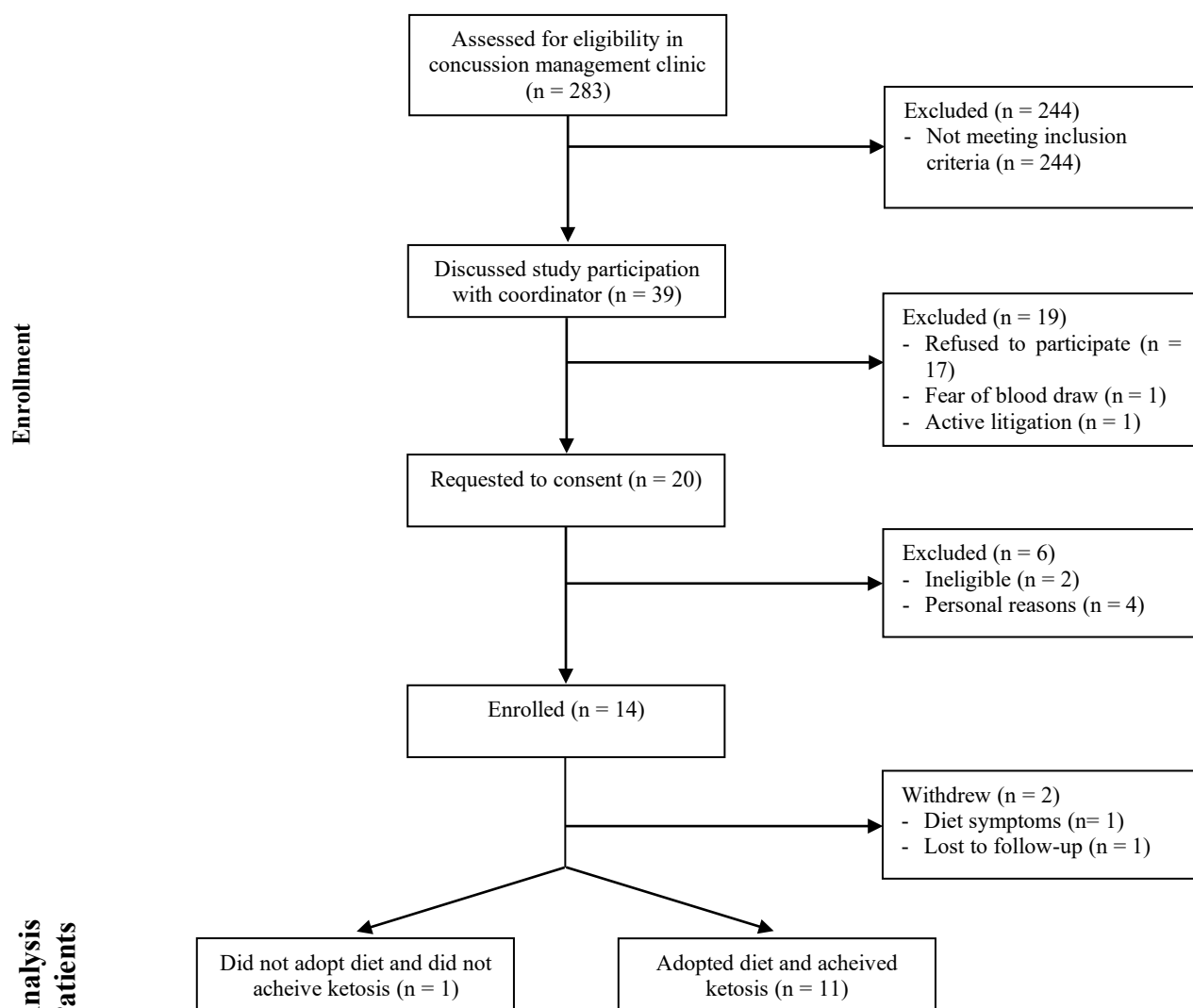

Supplement: Supplementary file 1 [file Image_1.pdf]
